# Update in Obstructive Sleep Apnea Syndrome in Children

**DOI:** 10.1016/S1808-8694(15)31288-X

**Published:** 2015-10-20

**Authors:** Aracy P.S. Balbani, Silke A.T. Weber, Jair C. Montovani

**Affiliations:** 1 Voluntary Professor, Ph.D., Discipline of Otorhinolaryngology and Head and Neck Surgery, Medical School Botucatu (UNESP); 2 Assistant Professor, Discipline of Otorhinolaryngology and Head and Neck Surgery, Medical School Botucatu (UNESP); 3 Full Professor, Discipline of Otorhinolaryngology and Head and Neck Surgery, Medical School Botucatu (UNESP)

**Keywords:** obstructive sleep apnea, child, learning, cognition

## Abstract

The prevalence of OSAS in children is 0.7-3%, with peak incidence in pre-schoolers. It is characterised by partial or complete upper airway obstruction during sleep, causing intermittent hypoxia. Both anatomical (severe nasal obstruction, craniofacial anomalies, hypertrophy of the pharyngeal lymphoid tissue, laryngeal anomalies, etc.) and functional factors (neuromuscular diseases) predispose to OSAS during childhood. The main cause of OSAS in children in adenotonsillar hypertrophy. The most common clinical manifestations of OSAS are: nocturnal snoring, respiratory pauses, restless sleep and mouth breathing. Nocturnal pulse oximetry, nocturnal noise audio/videotape recording and nap polysomnography are useful tools for screening suspected cases of OSAS in children, and the gold-standard for diagnosis is overnight polysomnography in the sleep laboratory. On the contrary of SAOS adults, children usually present: less arousals associated to apnea events, more numerous apneas/hypopneas during REM sleep, and more significant oxihemoglobin dessaturation even in short apneas. The treatment of OSAS may be surgical (adenotonsillectomy, craniofacial abnormalities correction, tracheostomy) or clinical (sleep hygiene, continuous positive airway pressure – CPAP).

## INTRODUCTION

Children may present several respiratory disturbances while sleeping: primary snoring, upper airways resistance syndrome and apnea/hypoapnea (central, obstructive or mixed apnea)^1^.

*Primary snoring* is characterized by loud upper airways breathing noise, but sleep frame, alveoli ventilation and hemoglobin oxygen saturation are sustained at normal levels. It is common in childhood, affecting from 7 to 9% of children aged 1 to 10 years^1^.

*Upper airways resistance syndrome* (*UARS*) is characterized by: night snoring, frequent EEG awakenings, fragmented sleep and increased upper airways resistance during inhaling air flow, but without significant flow reduction or oxyhemoglobin desaturation. Prevalence in children still remains unknwon^2^.

*Central apnea* is the interruption of the CNS (Central Nervous System) command to respiratory muscles resulting in cessation airflow at the nostrils and mouth and absence of respiratory effort, or chest/abdominal movements. It has higher prevalence in newborn and premature infants and it is also considered normal if does not affect arterial oxygen saturation (SpO2)^3^. *Obstructive Apnea* is the cessation of airflow at the nostrils and mouth due to collapse of upper airways, regardless of the effort of chest/abdominal muscles. In healthy children it is a rare event while they are sleeping and frequently does not last more than 10 seconds^4^. *Mixed Apnea* involves both decreased central respiratory control and upper airways obstruction. *Hypoapnea* is the partial reduction of airflow at the nostrils and mouth and can also be classified as central, obstructive or mixed^4^.

The Obstructive Sleep Apnea Syndrome (OSAS) was first described in children by William Osler in1892, but his study became systematized only after the 70's^3^. According to the International Classification of Sleep Disorders, OSAS is an “intrinsic sleep disorder characterized by frequent episodes of upper airways obstruction induced by hemoglobin dessaturation”^5^.

The prevalence of OSAS in children ranges from 0.7% to 3% in different epidemiological studies^5^, ^6^. The incidence peak was found in pre-school children within the age in which tonsil hypertrophy and adenoid are more common^2^. OSAS could have severe consequences as follows: *cor pulmonale*^3^, low body weight index^7^, behavioral problems, poor learning performance in school, and neurocognitive functions may be affected^8^, ^9^.

### Etiology of OSAS

Upper airways are permeable, under physiological conditions, due to anatomical and functional factors. Any abnormalities in such factors may trigger OSAS^3^.

### Anatomical Factors

#### 1. Bone abnormalities

Craniofacial bone is the scaffolding that protects the upper airways. Therefore, any malformation such as choanal atresia, micrognathia, mandibular hypoplasia and other skull base abnormalities (e.g. platibasia) may cause respiratory obstruction^10^.

Micrognathia is reported in more than 60 genetic syndromes (hemifacial microssomia, Treacher-Collins, Goldenhar, Pierre Robin, etc.) and frequently it coexists with microglossia and other malformations resulting in dorsal displacement of the tongue and oropharynx narrowing^11^.

In syndromes of craniofacial anomalies (Down, Crouzon, Apert, Pfeiffer, etc.) the skull base deformity and maxillary hypoplasia cause obstruction of nasal and rhinopharynx cavities^11^.

Bone abnormalities of rhinopharynx limits may decrease its antero-posterior diameter. Possibly it is the cause why not all children with adenoid hypertrophy have respiratory disorders while sleeping, however, some other children continue snoring even after adenoidectomy^10^.

Genioglossal bone promotes tongue protrusion preventing it from moving towards the posterior wall of the oropharynx under normal conditions. Mouth breathing habit results in dorso-caudal rotation of mandible changing the position of the genioglossal muscle by decreasing tongue protrusion^10^.

Abnormal dorsal-caudal positioning of the hyoid bone in some children is the cause of respiratory disorder while sleeping. These patients tend to sleep in cervical hyperextension position in which hyoid is elevated providing temporary relief of the obstruction^10^.

#### 2. Soft tissue abnormalities

Severe nasal obstruction due to rhinitis, tumors or bulky nasal polyps may cause mouth breathing and OSAS^12^.

In the first year of life, the larynx is more cranial and the glottis may reach soft palate. This configuration offers increased protection against food aspiration if the child makes sucking movement, however it facilitates pharynx obstruction^13^. Children with laryngomalacia and other laryngeal diseases (webs, tumors) may also have obstructive apnea.

The most common anatomical abnormality in children with OSAS is pharyngeal lymphatic tissue hypertrophy^14^, which occurs mainly from 3 to 8 years of age. It is worth pointing out that this abnormality seems to be a necessary factor, but it is not enough to trigger OSAS, since a) not all children with tonsil and adenoid hypertrophy have apnea, b) most children with palatine tonsils hypertrophy do not present respiratory obstruction while they are awake, when muscle tonus is increased, and c) many children that have undergone adenotonsillectomy present obstruction symptoms again in adolescence^3^. These facts make us believe that OSAS may result from a combination of anatomical and functional abnormalities in certain children^3^.

### Functional Factors

Hypotonia of intercostals and dilating muscles of the upper airways while sleeping. In REM sleep the activating reflex of the genioglossal and soft palate tensor muscles is reduced or absent resulting in decreased upper airways caliper, increasing its airflow resistance^13^.

Harvey et al.^15^ evaluated likely pre- and peri-natal OSAS-related factors in 40 children without neurological disorders. They reported increased incidence of complications during pregnancy (infections, need to be admitted to hospital, etc) in mothers of OSAS children compared to mothers of healthy children. Children with OSAS also tended to suffer more peri-natal complications such as hypoxemia and respiratory diseases. The authors analyzed the hypothesis of motherhood complications and peri-natal events, given that decreased neural control of the upper airways may be a predisposition to OSAS.

Children with neuromuscular diseases causing generalized hypotonia (muscular dystrophy) or incoordination (cerebral palsy) show increased risk of presenting severe OSAS^13^.

### Clinical Symptoms of OSAS

#### 1. Cardiovascular System

Marcus et al.^16^ studied 41 children with OSAS and found that 32% had systolic and diastolic BP (Blood Pressure) above 95th percentile, both while sleeping and awake. Hypertension was directly related to severity of obstructive apnea and level of obesity in children. The authors attributed the raise of blood pressure of these children to sub-cortical awakening, and not to hypoxemia, since there was not any relation found between pressure measurements and oxymetry. The authors pointed out the need to evaluate if OSAS treatment would normalize those children BP levels.

Upper airways obstruction and chronic alveolar hypoventilation result in abnormal lung ventilation/perfusion relation. Hypercapnia and hypoxemia cause respiratory acidosis and subsequent vasoconstriction of pulmonary artery increasing work load of right ventricle. At the same time, small and average caliper pulmonary arteries present remodeling and hypertrophy of smooth muscular layers that with time may evolve to myocardium hypertrophy and in some cases to right-ventricle dilation, heart failure and *cor pulmonale*^17^.

#### 2. Growth and metabolism

There are three theories to explain the occurrence of low weight index in many children with OSAS: 1. decreased production of growth hormone, 2. decreased caloric intake due to anorexia/dysphagia in children with adenoid tonsil hypertrophy, and 3. increased energetic spending due to nocturnal respiratory effort^18^.

Growth hormone (GH) is secreted during deep stages of REM sleep. Supposedly nocturnal secretion of GH is decreased in OSAS patients. Actually, blood level of “insulin growth factor” 1 (IGF-1), major GH-effect mediator, and “insulin growth factor binding protein” (IGFBP-3) are lower in OSAS children than in healthy children. After undergoing adenotonsillectomy these levels become normal.^18^.

Eva et al.^19^ believe that sympathetic discharge caused by mild apnea episodes result in increased serum catecholamine, cortisol and insulin concentrations. Studying the metabolism of 62 obese children and adolescents with OSAS they found that fast glycemia was abnormal in 11% of the cases, directly related to the apnea and hypoapnea index (IAH) recorded in polysomnography.

#### 3. Cognitive, learning and behavioral functions

Excessive day sleepiness (EDS) is one of the major complaints of adult patients with OSAS since sleep is fragmented. This complaint is less common in children with OSAS due to reduced number of awakenings and relative preservation of sleep architecture. The test of multiple sleep latencies - Multiple Sleep Latency Test (MSLT) is one of the best methods to evaluate day sleepiness. Average sleep latency lower than 10 minutes is an indicator of excessive sleepiness. The MSLT study carried out in 54 children with OSAS (diagnosed with an apnea index of >2) presented 14 children with primary snoring and 24 health control patients with sleep latency lower in the first test, however only 13% out of the total of OSAS children presented latency lower than 10 minutes^20^.

Intelligence, memory and attention tests performed in 16 school children referred to snoring treatment presented poor cognitive performance if compared to 16 control healthy patients of the same age. Children with snoring symptoms presented intelligence and attention deficit even without daytime sleepiness. It is assumed that attention deficit affects information processing and registering, decreasing learning ability in children with OSAS^21^.

A survey evaluated the prevalence of respiratory disorders while sleeping in 782 children with poor performance in first grade of primary public schools in the USA. Parents answered a questionnaire about respiratory symptoms of their children; subsequently, children underwent pulse oxymetry and capnography during the night. The prevalence of primary snoring in this sample was 22.2%, and sleep respiratory disorders was 18.1%.

Twenty-four children with respiratory sleep disorders have undergone adenotonsillectomy, and their school grades showed significant improvement in the year after surgery^9^.

Approximately 28% of the children with adenoid tonsillar hypertrophy presented behavioral changes such as aggressiveness and hyperactivity^2^. The prevalence of nocturnal snoring, however, common in 143 children with attention deficit and hyperactivity disorder (ADHD) was 30%^8^. Usually ADHD is treated with drugs and psychiatric support, but the pediatrician should be aware of the coexistence of respiratory disorders in children's sleep.

### OSAS Diagnosis

#### 1. Patient's history

Major OSAS symptoms in children include nocturnal snoring, respiratory pauses, labored breathing, restless sleep, night sweats and mouth breathing, but rhinitis, enuresis, sleeping in cervical^22^.

#### 2. Physical Examination

Li et al.^23^ pointed out that only the upper portion of palate tonsils is visible in oropharyngoscopy and may provide a false impression of its size and shape. The authors suggested profile radiography of upper airways for a more precise evaluation of air column and palatine tonsil obstruction.

Nasofibroscopy is useful in nasal cavity and rhinopharynx examination allowing the otorhinolaryngologist to diagnose nasal septum deviation and hypertrophy of nasal conchae and adenoid, as well as changes in respiratory dynamics and swallowing due to palatine tonsil hypertrophy.

Early diagnosis of OSAS in children have reduced findings of clinical symptoms of *cor pulmonale*^22^.

#### 3. Polysomnography

Polysomnography (PSG) performed in sleep laboratory during the entire night is the gold standard diagnostic method of OSAS. The exam has excellent repeatability, provide evidence of upper airways obstruction and differentiate obstructive apnea from central apnea, and records epileptic episodes in children with neurological disorders.^22^. Recommendations of “American Thoracic Society”^24^ for performing PSG in children are as follows:

1. Differential diagnosis between primary snoring and obstructive sleep apnea syndrome;
2. Evaluation of child with pathological sleep patterns (excessive daytime sleepiness, p. ex.);
3. Diagnostic confirmation of respiratory obstruction during sleep for surgical treatment recommendation;
4. Pre-operative evaluation of risks of respiratory complications of adenotonsillectomy or other surgeries of the upper airways;
5. Evaluation of laryngomalacia in patients since symptoms are more intense during nighttime or in presence of *cor pulmonale*;
6. Evaluation of obese children with excessive daytime sleepiness, snoring, polycithemia or *cor pulmonale*;
7. Evaluation of children with sickle cell anemia (due to risk of vascular occlusion during sleep);
8. Recurrence of snoring in adenotonsillectomy post-operatively;
9. Periodic control treatment of continuous airway pressure (CPAP).

There are many differences in children and adult OSAS found in polysomnography. In adult OSAS the apnea episode is almost always followed by cortical awakening resulting in fragmented sleep, but only 20% of children with OSAS presented cortical awakening^3^, ^13^, ^25^.

Most awakenings, obstructive apnea and hypoapnea in children occur during REM sleep. This characteristic is different in adults in which upper airways obstruction is more common in non-REM sleep^13^, ^25^.

Adult apnea is considered if the airflow cessation episodes last for 10 seconds or more. In this time interval an adult presents only two or three respiratory cycles, however small children can present up to six cycles due to their increased respiratory rate^3^.

Children suffer significant hemoglobin desaturation even in short-term apnea; they have higher metabolism and oxygen consumption than adults. Adults present total and cyclic obstruction of upper airways whereas children – especially those bellow three years of age- tend to have a long lasting partial obstruction, known as obstructive hypoventilation.^13^

In face of such differences, the adult PSG analysis parameters are inadequate for children. The “American Thoracic Society”^26^ recommends the following criteria:

• *Apnea Index (AI)*: Number of obstructive and mixed apnea episodes with minimal interval of two respiratory cycles. Expressed in episodes/per hour (considering the calculation of total sleeping time). OSAS is diagnosed in children if IA>1/hour.
• *Obstructive Hypoapnea*: Fifty percent (50%) air flow reduction or more associated with oxyhemoglobin desaturation >4%, or SaO_2_<90% and/or awakening.
• *Apnea-Hypoapnea Index* (IAH): The summation of the number of obstructive and mixed apnea, and hypoapnea. Expressed in episodes per hour (considering the calculation of total sleep time). The abnormal diagnostic for children would be IAH>1/hour. The AIA is also known as Respiratory Disorder Index (RDI)^14^.

Harvey et al.^15^ classified OSAS in children as mild (1>IAH<5/hour), *moderate* (5>IAH<9/hour and *severe* IAH>10/hour.

*Hemoglobin O_2_ Saturation* (O_2_Sa): consider minimal saturation (nadir O_2_Sa) and average oxygen saturation during diagnostic test. Diagnostic of O_2_ Sa<90% nadir is associated with obstructive apnea.

*Alveolar Hypoventilation*: Total sleep time with hypercapnia percentage is calculated (CO_2_>50mmHg) at the end of expiration. Hypoventilation is considered if CO_2_>50mmHg occur at the end of expiration for more than 8% of total sleep time or variation of CO_2_>13mmHg related to basal value.

Children with sleep time respiratory grasp, but IAH <1, absence of hemoglobin desaturation or hypercapnia during PSG are diagnosed as primary snoring.

#### 4. Other diagnostic tests

The insufficient number of pediatric sleep laboratories to meet the PSG demand, as well as the high cost of the exam encouraged the use of other diagnostic methods.

One or two hour daytime *PSG* (“nap polysomnography”) was suggested for screening suspect OSAS cases. The method has the following disadvantages: 1. differently from nighttime PSG, daytime test commonly required sedation that may increase obstructive apnea episodes; 2. during daytime PSG children may not be REM sleep and respiratory obstruction episodes are more common exactly during in this sleep phase^26^. Therefore daytime PSG tends to underestimate obstructive apnea and hypoapnea episodes^25^.

According to the American Pediatric Association^22^, screening PSG is valuable if it detects apnea and hypoapnea. In clinical suspicion of OSAS, even in case of normal daytime PSG, children should undergo nighttime PSG.

In face of children's difficulty in adapting to sleep laboratory and in order to provide some comfort for the parents, Goodwin et al.^27^ performed 157 *PSG tests* in home settings in children from 5 to 12 years. Fifteen tests were not satisfactory because pulse oxymeter got out from the child's finger in the middle of the night, the child did not cooperate or due to technical issues (disconnected cable, battery failure in the equipment). Only 61% of the findings were considered of excellent quality. Therefore the suggestion is to perform PSG at patient's home only in case patient's is clinically unable to go to the sleep laboratory.

Brouillette et al.^28^ evaluated the results of night oxymetry of 349 children submitted to PSG with suspicion of OSAS due to adenotonsillar hypertrophy. Oxymetry data were analyzed without the knowledge of PSG results. Oxymetry was considered positive for OSAS with three or more episodes of desaturation were recorded in a 10-30 minute interval with at least three events resulting in O2SA<90%. In 93 children with positive oxymetry, ninety had OSAS diagnostic confirmed in PSG (IAH>1). In the present study, the positive predictive night pulse oxymetry value for the diagnostic of OSAS was 97%. The authors pointed out that normal oxymetry in cases of clinical OSAS suspicion does not rule out the syndrome and child should be referred to PSG.

*Audio taping respiratory noises* of nighttime sleep is also used to screen suspect cases of OSAS. It may be recorded by the parents in the child's sleeping room. The analysis of the audiotape is performed by a medical team to assess the intensity of snoring and respiratory pauses^29^. The sensitivity of the method in detecting apnea is higher than 90%, but its predictive value does not exceed 50%^22^.

*Videotaping child's sleeping images* is expensive and technically complicated and may require the use of infrared cameras to videotape low light settings. The analysis of the child's respiratory pattern has 94% sensitivity for the diagnostic of OSAS with positive predictive value of 83%^22^.

*Rx cephalometry of upper airways profile* analyses craniofacial structures and measure airway column in different points which is particularly important in children with malformations.^10^

### OSAS Treatment

#### 1. Surgical Treatment

Adenotonsillectomy allows the cure of OSAS in 75-100% of children with adenotonsillar hypertrophy^7^.

The risk of respiratory complications in postoperative adenotonsillectomy is higher in case the surgery is recommended for OSAS than in recurrent pharyngotonsillitis. High risk children in terms of respiratory complications are: nursing infants, infants with craniofacial anomalies, Down Syndrome and other genetic disorders, cerebral palsy, neuromuscular diseases, metabolic and accumulation disturbances, chronic obstructive lung disease, *cor pulmonale* and sickle cell anemia^22^, children with O_2_Sa<70% or IAH>10/hour^26^. The American Pediatric Association recommends in such cases that children remained in hospital the night after the surgery to have continuous monitoring of pulse oxymetry^22^.

Although tracheostomy is effective to treat OSAS, the procedure may have some complications (stoma stenosis, canulla obstruction by secretion plugs or granulation tissue formation) and it increases the need for family care provided to patient. Therefore, a specific treatment is advocated to treat obstruction points in upper airway (otorhinolaryngological, orthognathic and maxillofacial surgeries)^11^.

Twenty-five surgeries to treat micrognathia were surveyed and were successful in relieving respiratory obstruction in 23 cases, reoperation was required in two cases. Eight percent of the children were able to have the tracheostomy closed post-operatively^11^.

Uvulectomy and uvulopalatopharyngoplasty might be performed jointly with adenotonsillectomy to treat OSAS, depending on evaluation of ENT and members of multidisciplinary team.

These procedures are applicable in neuromuscular diseases and in cerebral palsy.

#### 2. Clinical Treatment

Continuous Positive Airway Pressure (nasal CPAP or BiPAP) is recommended if: 1. absence of adenotonsillar hypertrophy, 2. surgical apnea treatment is not indicated; 3. persistent OSAS after surgical treatment^22^. A new PSG test is recommended six to eight weeks post-operatively to confirm OSAS^13^ persistence. The CPAP may also be used temporarily in severe OSAS associated with genetic syndromes, mucopolysaccharidosis and cerebral palsy until the child is ready for the surgery^13^.

Nasal CPAP has not been approved by “Food and Drug Administration” to be used in children weighting less than 30kg^13^.

The guidelines for sleep hygiene, obesity treatment and rhinitis are also important in treatment approach of OSAS^22^ children. Chronic effects of mouth breathing need to be corrected with joint multidisciplinary effort with speech and/or orthodontic therapy to reestablish normal breathing patterns and craniofacial growth^30^.

## DISCUSSION

Studies about children sleeping respiratory disorders have been increasingly developed over the last decades. There are, however, some diagnostic and treatment imbalances of the diseases in clinical practice.

According to Bower, Buckmiller^2^ the delay in diagnostic of OSAS is common in children of underdeveloped countries. The gap between onset of symptoms and the diagnostic may reach three years^2^, increasing risks of cardiovascular and metabolic complications, as well as impairment of cognitive and school-learning functions^8^, ^9^. Meanwhile, the overall family's quality of life is worsened due to respiratory anguish and discomfort of the child during sleep.

The gold standard diagnostic test for OSAS is nighttime polysomnography (PSG) in a sleep laboratory^22^, ^24^. The “American Thoracic Society” recommends that PSG should be performed whenever diagnostic confirmation is required to evaluate surgical risk of children in undergoing adenotonsillectomy and to control clinical and surgical treatment of OSAS^24^. In our country, however, the number of care centers qualified to perform PSG is far from the ideal demand resulting in the need for bringing patients to large centers in which the waiting list of patients is long. Other aggravating factors are: PSG high cost in private sleep clinics and its exclusion from procedures to be reimbursed by health insurance companies. Therefore, although ideally we should confirm OSAS diagnosis in all suspected cases, in practice most of the children that do not have additional health symptoms might undergo adenotonsillectomy based on the evaluation of the Pediatrician and Otorhinolaryngologist and on radiograph or nasofibroscopy findings.

If there are other associated diseases (craniofacial malformation, heart, neurological diseases, etc) all possible efforts should be made to perform PSG in sleep laboratory to evaluate the severity of apnea and the risk of respiratory complications in adenotonsillectomy post-operatively.^22^. If the diagnostic test is really not available, the doctor could alternatively perform night pulse oxymetry and audio taping of respiratory noise while the child is sleeping^28^, ^29^.

Regarding PSG we would like to point out that there is not any consensus on diagnostic criteria of OSAS and its severity measurement^24^, ^26^. Certainly some more years will be required to define the normal and pathological patterns of PSG in children from different age groups.

Literature reported that adenotonsillectomy allowed OSAS cure in more than75% of children with adenotonsillar hypertrophy with positive impact in their growth, behavior and cognitive functioning^9^. Several other surgical procedures to repair anomalies of the upper airways also present good results and sometimes it is possible to postoperatively remove tracheostomy Os^11^.

It is important to bear in mind that the otorhinolaryngologist should have special care in operating children with OSAS associated with other conditions (neurological, cardiovascular, malformations, craniofacial, etc.), since they present increased risk of respiratory complications post-operatively. Surgical procedures should be carried out in hospital settings with adequate devices and with ICU and Semi Intensive Care Units to properly monitor children post-operatively.

In spite of being costly, nasal CPAP or BiPAP therapy brings significant benefits to children with OAS associated with neurological or muscular diseases^13^ deserving to be better studied in nursing infants.

## CLOSING REMARKS

We would like to point out that the multidisciplinary team – pediatricians, neuropediatricians and otorhinolaryngologists- should be aware of the importance of early diagnostic and treatment of OSAS to prevent complications and to improve the child's quality of life.
